# Conjugation of Cholesterol to HIV-1 Fusion Inhibitor C34 Increases Peptide-Membrane Interactions Potentiating Its Action

**DOI:** 10.1371/journal.pone.0060302

**Published:** 2013-04-02

**Authors:** Axel Hollmann, Pedro M. Matos, Marcelo T. Augusto, Miguel A. R. B. Castanho, Nuno C. Santos

**Affiliations:** Instituto de Medicina Molecular, Faculdade de Medicina, Universidade de Lisboa, Lisbon, Portugal; German Primate Center, Germany

## Abstract

Recently, the covalent binding of a cholesterol moiety to a classical HIV-1 fusion inhibitor peptide, C34, was shown to potentiate its antiviral activity. Our purpose was to evaluate the interaction of cholesterol-conjugated and native C34 with membrane model systems and human blood cells to understand the effects of this derivatization. Lipid vesicles and monolayers with defined compositions were used as model membranes. C34-cholesterol partitions more to fluid phase membranes that mimic biological membranes. Importantly, there is a preference of the conjugate for liquid ordered membranes, rich in cholesterol and/or sphingomyelin, as observed both from partition and surface pressure studies. In human erythrocytes and peripheral blood mononuclear cells (PBMC), C34-cholesterol significantly decreases the membrane dipole potential. In PBMC, the conjugate was 14- and 115-fold more membranotropic than T-1249 and enfuvirtide, respectively. C34 or cholesterol alone did not show significant membrane activity. The enhanced interaction of C34-cholesterol with biological membranes correlates with its higher antiviral potency. Higher partitions for lipid-raft like compositions direct the drug to the receptor-rich domains where membrane fusion is likely to occur. This intermediary membrane binding step may facilitate the drug delivery to gp41 in its pre-fusion state.

## Introduction

The development of new drugs against the human immunodeficiency virus type 1 (HIV-1) has been the focus of intense research since its discovery [1]. The virus fusion with the cell membrane and the consequent entry into the host cell is a critical moment of its life cycle. Efficiently blocking this process prevents all the subsequent intracellular steps. Most importantly, the integration of the viral genome, which can stay silent for years, does not occur. Despite this promising approach, only two HIV entry inhibitors are available in the market: maraviroc, an inhibitor of envelope binding to the CCR5 co-receptor [2], and enfuvirtide, a fusion inhibitor peptide targeting gp41 in its pre fusion conformation [3]. Enfuvirtide, due to its peptide nature, has to be administered subcutaneously and is more sensitive to degradation while in circulation. It is important to overcome these limitations as peptide drugs can also have the advantage to be potentially less toxic.

Several HIV-1 fusion inhibitor peptides have been developed and studied *in vitro* [4]–[6]. The rationale for the development of these peptides is based on the structural rearrangements of gp41 after the binding of gp120 to CD4 and a co-receptor (CCR5 or CXCR4). The two heptad repeats fold into each other creating a hairpin structure (also named 6-helix bundle, due to the formation of a homotrimer of heterodimers) that brings the two membranes together and promotes the formation of the fusion pore used for the entry of the viral content into the target cell [7]. Most of these peptides were derived from the C-terminal heptad repeat (CHR) region of gp41, and hence bind to the opposite domain in order to prevent bundle formation. The rational design of higher efficacy peptides compared to the native sequence from gp41 CHR include rearrangements and substitutions in order to improve intrinsic characteristics such as solubility, helical stability, oligomerization, lipophilicity and resistance to proteolysis [4].

In our previous works, we emphasized the importance of the lipophilicity and membrane interaction properties of the fusion inhibitor peptides enfuvirtide, T-1249 and sifuvirtide [8]–[10]. The fact that these peptides can partition to the membranes and to the microdomains, where receptors are preferentially located, facilitates the interaction with gp41 in its exposed conformation. This intermediate is temporally and spatially constrained; thus, a pre-concentration effect in the membrane can be a strategy to overcome this poorly accessible target.

Previously, the peptide C34, a classical fusion inhibitor used also as a template for the structural characterization of gp41 [7], was conjugated with a cholesterol moiety in order to obtain higher efficacy and lifetime *in vivo* [11]. C34-cholesterol (with cholesterol covalently bound to the C-terminus of the peptide; also known as L’644) was shown to be 50-fold more potent than C34 alone in terms of IC_50_ (HXB2 strain). Moreover, in a mouse model, the conjugated peptide was still detected 24 h after injection and with higher concentration, when compared with the unconjugated peptide, which was undetectable after 6 h [11]. In another recent work, a comparative pre-clinical evaluation of four fusion inhibitors (C34, T1249, enfuvirtide and C34-cholesterol) as potential microbicides showed that C34-cholesterol exhibits the highest anti HIV-1 activity as well as an extended window of activity. Moreover, C34-cholesterol is able to inhibit both wild type and reverse transcriptase inhibitors-resistant isolates [12].

The conjugation approach to improve fusion inhibitor peptides, such as by attaching acyl chains of various sizes [13]–[15] or human serum albumin [16], [17], has been tried before; however, none has reached beyond pre-clinical tests. C34-cholesterol is a promising candidate [12].

In this article, we aimed at characterizing the interaction of C34 and C34-cholesterol with biomembranes to elucidate the peptide-membrane interaction and possible relations with the efficiency of these drugs. Our approach first covered a detailed study of the peptide partition towards biomembrane model systems, namely large unilamellar vesicles (LUV), by following the intrinsic fluorescence changes of the peptide tryptophan residues, located at the N-terminal region, when it adsorb or penetrate in the lipid environment. This methodology allows testing a diversity of lipid compositions, in order to dissect the role of the different lipid types. Insertion of the fusion inhibitors in lipid monolayers was also evaluated on a Langmuir-Blodgett trough. Finally, we studied the interaction with membranes of human blood cells, in an *ex vivo* setting. In this case, we used a lipophilic fluorescent probe (di-8-ANEPPS) that is sensitive to the membrane dipole potential and can report interactions of molecules that disturb the membrane order.

## Experimental Section

### Reagents

C34 (WMEWDREINNYTSLIHSLIEESQNQQEKNEQELL) was obtained from the NIH AIDS Research and Reference Reagent Program (Division of AIDS, NIAID, NIH). C34-cholesterol (L’644) was a kind gift from the International Partnership for Microbicides (licensed by Merck) and its sequence is C34-GSGC-Cholesterol. 5NS (5-doxyl-stearic acid) and 16NS (16-doxyl-stearic acid) were from Aldrich (Milwaukee, WI, USA). L-Tryptophan, acrylamide, HEPES and NaCl were from Merck (Darmstadt, Germany). POPC (1-palmitoyl-2-oleoyl-*sn*-glycero-3-phosphocholine), DPPC (1,2-dipalmitoyl-*sn*-glycero-3-phosphocholine), POPG (1-palmitoyl-2-oleoyl-*sn*-glycero-3-[phospho-*rac*-(1-glycerol)]), DHSM (N-dodecanoyl-sphinganine-1-phosphocholine), SM (egg sphingomyelin), POPE (1-palmitoyl-2-oleoyl-*sn*-glycero-3-phosphoethanolamine) and POPS (1-palmitoyl-2-oleoyl-*sn*-glycero-3-phospho-L-serine) were purchased from Avanti Polar Lipids (Alabaster, AL, USA), while cholesterol (Chol) were from Sigma (St. Louis, MO, USA).

### Fluorescence Spectroscopy Measurements

C34 and C34-cholesterol peptides contain tryptophan residues, which make fluorescence techniques suitable tools to probe these molecules. The working buffer used throughout the studies was HEPES 10 mM pH 7.4 in NaCl 150 mM. C34 (150 µM) and Trp (500 µM) stock solutions were prepared in buffer. C34-cholesterol (500 µM) stock solutions were prepared in DMSO. Large unilamellar vesicles (LUV) were prepared by extrusion methods, as described elsewhere [18], [19].

Membrane partition and fluorescence quenching studies using acrylamide were carried out in a Varian Cary Eclipse fluorescence spectrophotometer (Mulgrave, Australia) and time-resolved fluorescence spectroscopy studies in a LifeSpec II Fluorescence Lifetime spectrometer (Edinburgh Instruments).

The fluorescence spectral characterization of C34, C34-cholesterol and Trp was performed with an excitation wavelength of 280 nm, except for 5NS, 16NS and acrylamide quenching experiments, where the excitation was performed at 290 nm to minimize the relative quencher/fluorophore light-absorption ratios. For the quenching experiments, fluorescence emission was collected at a fixed wavelength of 350 nm and for the partition studies integrated spectra from 310 to 450 nm were used. Typical spectral bandwidths were 5 nm for excitation and 10 nm for emission. Excitation and emission spectra were corrected for wavelength-dependent instrumental factors [20]. During the quenching and partition experiments, emission was also corrected for successive dilutions, scatter [21] and simultaneous light absorptions of quencher and fluorophore. All the fluorescence measurements in this study were performed at room temperature (approximately 25°C).

### Partition Coefficient Determination

Membrane partition studies were performed by successive additions to a 5 µM C34 or C34-cholesterol solution of small amounts of LUV suspensions with different lipid compositions, including: POPC, POPC:Chol (2∶1 and 1∶1), DPPC, POPC:Chol:SM (1∶1:1 and 2∶2:1), POPC:SM (2∶1), POPC:POPG (5∶1), POPC:DHSM (1∶0.06), and HIV membrane-like mixture with and without DHSM (POPC 5.3%, DPPC 3.5%, cholesterol 45.3%, SM 18.2%, POPE 19.3% and POPS 8.4% [22]), with a 10 min incubation time between each addition. The partition coefficients (*K*_p_) were calculated using the equation [23]:
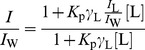
(1)where *I*_W_ and *I*_L_ are the fluorescence intensities in aqueous solution and in lipid, respectively, *γ*_L_ is the molar volume of the lipid [24], [25], and [L] its concentration.

### Surface Pressure

Changes on the surface pressure of lipid monolayers induced by C34, C34-cholesterol, enfuvirtide and T-1249 were measured in a Langmuir-Blodgett trough NIMA ST900 (Coventry, UK), at constant temperature (25±0.5°C). The surface of a HEPES buffer solution contained in a Teflon trough of fixed area was exhaustively cleaned by surface aspiration. Then, a solution of lipids in chloroform was spread on this surface, reaching a surface pressure of 20.5±1 mN/m. Peptide solutions were injected in the subphase and the changes on the surface pressure were followed during the necessary time to reach a constant value. The surface pressure of an air−water interface upon injecting the largest concentration of each peptide used throughout the studies was always below 15 mN/m (data not shown). For this reason, the lowest initial surface pressure of the lipid monolayers before the addition of the peptides to the subphase was above that value. In this condition, the changes in surface pressure observed upon the injection of the peptide can be attributed to an effect of the peptide on the monolayer interfacial tension.

The dissociation constant (*K*_d_) was calculated from the adsorption Langmuir isotherm:
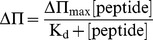
(2)where ΔΠ are the changes of surface pressure, ΔΠ_max_ is the maximum change of pressure achieved and [*peptide*] is the peptide concentration.

### Acrylamide Quenching

The fluorescence quenching of 5 µM C34 or C34-cholesterol by acrylamide (0−60 mM) [8] was studied in buffer and in the presence of POPC or DPPC 3 mM LUV, by successive additions of small volumes of the quencher stock solution. For every addition, a minimal 10 min incubation time was allowed before measurement. Quenching data were analyzed by using the Stern−Volmer equation [23]:
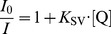
(3)where *I* and *I*_0_ are the fluorescence intensity of the sample in the presence and absence of quencher, respectively, *K*_SV_ is the Stern−Volmer constant and [Q] the concentration of quencher.

### 5NS and 16NS Quenching

Fluorescence quenching assays with the lipophilic probes 5NS and 16NS were performed by time-resolved fluorescence spectroscopy. These assays were carried out at the same peptide and lipid concentrations used for the acrylamide quenching, by successive additions of small amounts of these quenchers (in ethanol) to samples of peptide in POPC and POPC:Chol (2∶1), keeping the ethanol concentration below 2% (v/v) [26]. The effective lipophilic quencher concentration in the membrane was calculated from the partition coefficient of both quenchers to the lipid bilayers [27]. For every addition, a minimal 10 min incubation time was allowed before measurement. Quenching data were analyzed by using the Stern−Volmer equation (eq. 1), or the Lehrer equation [27], [28] when a negative deviation to the Stern–Volmer relationship is observed:
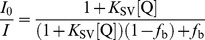
(4)where *f*_b_ is the fraction of light arising from the fluorophores accessible to the quencher.

In the case of dynamic quenching, the relationship *I*_0_ */I* = *τ*_0/_ *τ* is valid; thus, time-resolved quenching data can be analyzed by using the same equations (eq. 3 and 4).

### Membrane Dipole Potential Assessed by di-8-ANEPPS

Human blood samples were obtained from healthy volunteers, with their previous written informed consent, at the Instituto Português do Sangue (Lisbon, Portugal). This study was approved by the ethics committee of the Faculdade de Medicina da Universidade de Lisboa. Isolation of erythrocytes and PBMC and labeling of these cells with di-8-ANEPPS (Invitrogen, Carlsbad, CA, USA) were performed as described before [9], [29]. For erythrocytes isolation, blood samples were centrifuged at 1200 × *g* during 10 min, plasma and buffy-coat were removed, and remaining erythrocytes were washed twice in working buffer. They were incubated at 1% hematocrit in buffer supplemented with 0.05% (m/v) Pluronic F-127 (Sigma) and di-8-ANEPPS 10 µM. PBMC were isolated by density gradient using Ficoll-Paque Plus (GE Healthcare, Little Chalfont, UK) and counted in a Neubauer improved hemocytometer. They were incubated at 3000 cells/µL in Pluronic-supplemented buffer with 3.3 µM di-8-ANEPPS. Cells were incubated with the fluorescent probe during 1 h, with gentle agitation, and unbound probe was washed with Pluronic-free buffer on two centrifugation cycles. C34, C34-cholesterol (in DMSO stock solution) or cholesterol (in DMSO:ethanol 1∶1 stock solution) were incubated with erythrocytes at 0.02% hematocrit and with PBMC at 100 cells/µL during 1 h, with gentle agitation, before the fluorescence measurements. For lipid vesicles labeling, suspensions with 500 mM of total lipid were incubated overnight with di-8-ANEPPS 10 µM, to ensure maximum incorporation of the probe. The maximum concentration of DMSO or DMSO:ethanol in the suspensions was 2.4% (v/v) at 6 µM of peptide or cholesterol. Excitation spectra and the ratio of intensities at the excitation wavelengths of 455 and 525 nm (*R* = *I*_455_/*I*_525_) were obtained with emission set at 670 nm to avoid membrane fluidity-related artifacts [30], [31]. Excitation and emission slits for these measurements were set to 5 and 10 nm, respectively. The variation of R with the peptide concentration was analyzed by a single binding site model [32]:
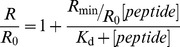
(5)with the *R* values normalized for *R*_0_, the value in the absence of peptide. *R*_min_ defines the asymptotic minimum value of *R* and *K*_d_ is the dissociation constant.

### Data Analysis

Fitting of the equations mentioned in this article to the experimental data was done by non-linear regression using Graphpad Prism 5. Error bars on data presentation represent the standard error of mean (SEM).

## Results

### Membrane Partition

**Figure 1:**
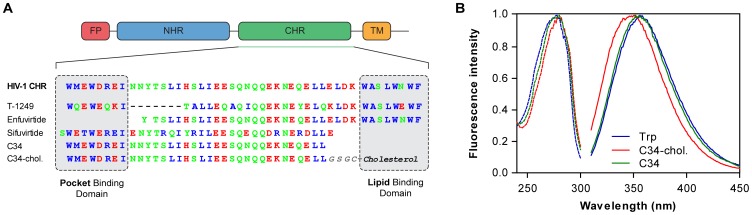
Characteristics of C34 and C34-cholesterol. (**A**) Schematic representation of HIV-1 gp41 main domains, depicting the relative position of the fusion peptide (FP), N-terminal heptad repeat domain (NHR), C-terminal heptad repeat domain (CHR) and transmembrane region (TM). The sequence of C34 was aligned with enfuvirtide, T-1249 and sifuvirtide, showing the pocket binding domain (PBD) and the lipid binding domain (LBD). Hydrophobic residues are represented in blue, non-charged polar residues in green, and charged polar residues in red. (**B**) Normalized fluorescence excitation (dashed line) and emission (solid line) spectra of 5 µM C34, C34-cholesterol or Trp in aqueous solution.

UV-visible absorption and fluorescence spectra in buffer of C34 are comparable to those of Trp in aqueous solution (Fig. 1); however, C34-cholesterol presents a blue shift, indicating that there is a change in the Trp surrounding environment in this conjugate.

**Figure 2:**
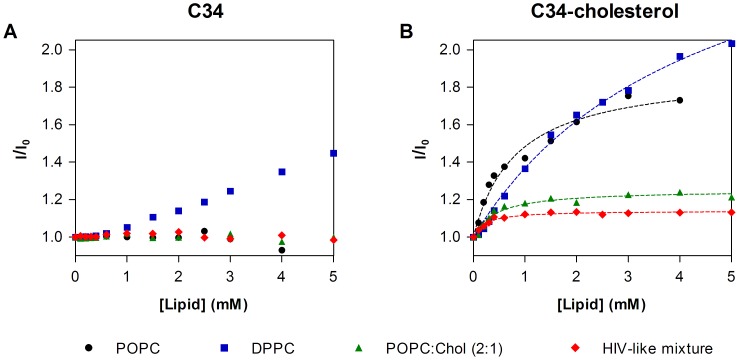
Partition of the peptides to lipid vesicles. Evaluation of Trp fluorescence variations of 5 µM C34 (**A**) or C34-cholesterol (**B**) upon titration with large unilamellar vesicles (LUV), performed by successive additions of POPC, POPC:Chol 2∶1, DPPC or HIV-like mixture LUV suspension. Dashed lines are fittings of eq. 1 to the experimental data.

As shown in Fig. 2, there is an increase in the fluorescence intensity of C34-cholesterol in the presence of LUV. The partition coefficient between the lipid and aqueous phases, *K*_p_, was determined by fitting eq. 1 to the fluorescence intensity data, in order to quantify the extent of interaction of the peptides to the LUV (Table 1). For POPC, a lipid with packing density and fluidity properties similar to biological membranes, a *K*_p_ value of 1.57×10^3^ was obtained, showing a significant interaction. When gel phase DPPC membranes were tested, a decreased *K*_p_ was found, probably due to the higher bilayer rigidity. In cholesterol-rich membranes (POPC:Chol, POPC:Chol:SM 2∶2:1 and HIV-like mixture) a significant increase in the partition was observed, when compared with pure POPC, also with a decrease on I_L_. Mixtures with cholesterol and SM mimic membrane microdomains usually named lipid rafts [33]. The same variation on I_L_ occurred for the addition of SM or DHSM (an unusual sphingolipid which does not contain a 4,5-*trans* double bond in its sphinganine backbone, significantly enriched in HIV-1 membranes [22]). The HIV-like mixture had the highest partition, hinting to an importance of the viral membrane in capturing the drug besides the cell membrane.

**Table 1:** Partition coefficients.

Lipid Mixture	*K*_p_	I_L/_I_w_
POPC	1574±261	1.88±0.05
DPPC	331±44	2.98±0.16
POPC:Chol 2∶1	3454±718	1.25±0.01
POPC:Chol:SM 2∶2:1	4050±885	1.24±0.01
POPC:Chol 1∶1	4612±436	1.32±0.01
POPC:DHSM (6%)	755±232	1.72±0.09
HIV-like mixture	8084±1190	1.14±0.004
HIV-like mixture with DHSM	1758±688	1.15±0.002

Parameters obtained from the fitting of the fluorescence data of the partition assays of C34-cholesterol 5 µM using eq. 1. All measures were made at least in triplicate. Values are presented as mean ± standard error of mean (SEM).

In the case of C34, except for DPPC, no significant changes in the fluorescence intensity were observed in the presence of membranes (Fig. 2A), indicating an absence of significant peptide-membrane interactions.

### Surface Pressure Perturbation of Monolayers

**Figure 3:**
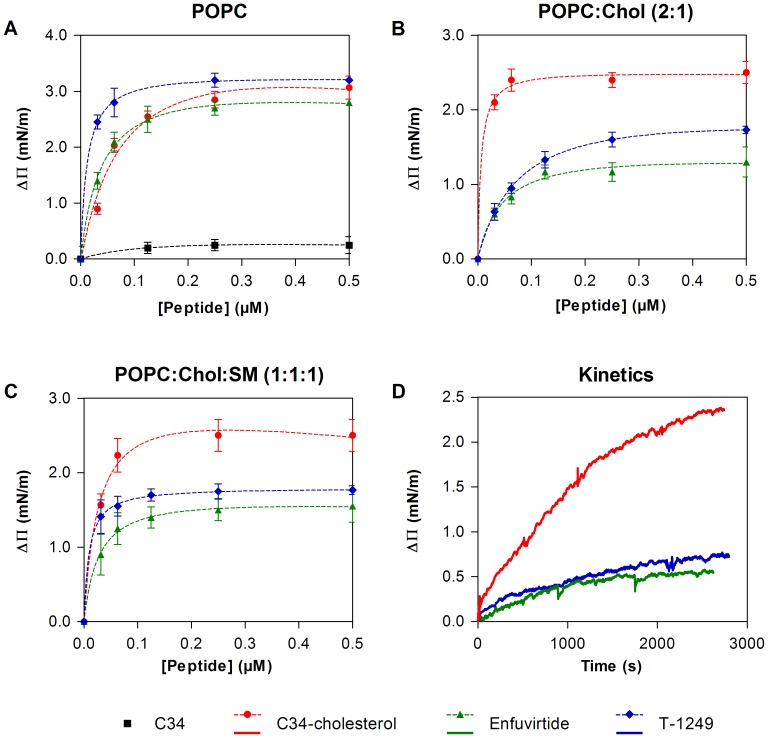
Interaction of the fusion inhibitor peptides with lipid monolayers. (**A−C**) Changes in surface pressure as a function of HIV-1 fusion inhibitor addition to pure POPC (**A**), POPC:Chol 2∶1 (**B**) or POPC:Chol:SM 1∶1:1 (**C**) monolayers. Dashed lines are fittings of eq. 2 to the experimental data. (**D**) Changes in surface pressure as a function of time after addition of HIV fusion inhibitors to achieve a final concentration of 31 nM on a POPC:Chol 2∶1 monolayer. All assays were carried out at 25°C, using an initial pressure of 21.5±0.5 mN/m. Each point is the average of at least triplicates of independent samples. Error bars represent the standard error of mean (SEM).

Despite the negligible partition and quenching results, adsorption of C34 to the membrane surface cannot be discarded, as it may leave the Trp residues exposed to the bulk aqueous environment, with unchanged quantum yield (*i.e.*, not contributing for *K*_p_ calculation) [10]. In order to discard that situation, surface pressure measurements on POPC monolayer were carried out, using a low initial surface pressure (20.5 mN/m). Usually, the adsorption and penetration of molecules is favored at low surface density (loosely packed lipid monolayers), whereas high surface pressure (compact lipid packing) hinders the adsorption and penetration in the lipid monolayers [34]. As shown in Fig. 3A, C34 is not able to induce any significant change in the surface pressure, in contrast to C34-cholesterol.

We also measured the changes in surface pressures induced by enfuvirtide and T-1249, yielding similar results to C34-cholesterol (Fig. 3A). In contrast, with POPC:Chol (2∶1) or POPC:Chol:SM (1∶1:1) monolayers, C34-cholesterol was able to induce larger changes than the other inhibitors (Figure 3B,C). *K*_d_ and ΔΠ_max_ values were determined by fitting eq. 2, in order to quantify the interaction of each peptide with the Langmuir monolayers (Table 2).

**Table 2:** Dissociation constants, *K*_d_, and ΔΠ_max_ determined from surface pressure changes.

	C34-cholesterol		Enfuvirtide			T1249		
	*K*_d_(nM)	ΔΠ_max_(mN/m)	*K*_d_(nM)		ΔΠ_max_(mN/m)	*K*_d_(nM)	ΔΠ_max_(mN/m)	
**POPC**	59±15	3.5±0.3	31±3.7	3.1±0.1		12±0.6	3.3±0.02	
**POPC:Chol (2∶1)**	5.3±1.4	2.5±0.05	41±6.8	1.4±0.06		65±3.5	1.9±0.03	
**POPC:Chol:SM (1∶1:1)**	9.2±1.3	2.6±0.02	27±4.6	1.6±0.03		13±1.1	1.8±0.02	

Parameters obtained from the fitting of the surface pressure data from Fig. 3 using eq. 2. All measures were made at least in triplicate. Values are presented as mean ± standard error of mean (SEM).

Direct comparison between the three peptides is difficult since they have different membrane binding domains. Therefore, they are not expected to modify the same way the surface tension of the membrane. However, comparing the same peptide for different lipids, we observe that C34-cholesterol presents an increased affinity to cholesterol rich membranes: an approximate ten-fold increase for POPC:Chol (2∶1) and around six-fold for POPC:Chol:SM (1∶1:1), when compared to pure POPC. This is in good agreement with data obtained from partition studies. In the case of enfuvirtide, no significant changes in the affinity were observed among each membrane composition tested, with just a slight decrease in the affinity for POPC:Chol, in a good agreement with previously published data [35]. Finally, T-1249 presented lower affinity for POPC:Chol, when compared with the other two compositions.

When the interaction kinetics were tested, a faster variation was observed for C34-cholesterol (Figure 3D). Control experiments were carried out using DMSO without C34-cholesterol and no significant changes were observed.

### Localization in the Lipid Bilayer

**Figure 4:**
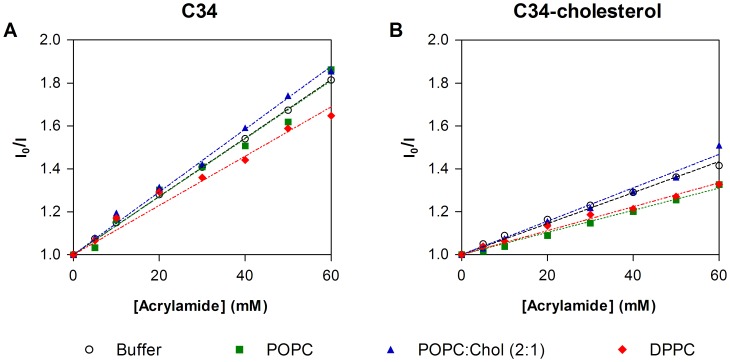
Accessibility of the peptide to the aqueous medium. Fluorescence quenching by acrylamide of C34 (**A**) and C34-cholesterol (**B**) in the presence (filled symbols) and absence (empty circles) of lipid vesicles (5 µM peptide and 3 mM total lipid). Lipid compositions tested were pure POPC, POPC:Chol 2∶1 and pure DPPC. Dashed lines are fittings of the Stern-Volmer equation (eq. 3) to the experimental data.

In order to test the accessibility of the fluorophores to the aqueous environment, the fluorescence quenching of the Trp residues of the peptide by acrylamide was studied. Linear Stern-Volmer plots were obtained in the absence of lipid (Fig. 4) and the quenching for C34-cholesterol is less efficient than C34, indicating that the fluorophore in the conjugate is less exposed to the surrounding aqueous medium. The cholesterol moiety may create hydrophobic pockets due to the formation of micro-aggregates, leading to this difference in the quenching in aqueous solution. Stern−Volmer plots and *K*_sv_ constants in the presence of 3 mM of lipid and 5 µM of C34 or C34-cholesterol were obtained in order to complement the partition assays (Fig. 4). For C34-cholesterol, both assays with lipids yielded *K*_sv_ values lower than the obtained in their absence (7.23±0.15 mM^−1^ in solution, 5.58±0.14 mM^−1^ with DPPC and 5.16±0.11 mM^−1^ with POPC). In contrast, for C34 only, DPPC was able to reduce the *K*_sv_ value from 13.6±0.01 mM^−1^ in solution to 11.5±0.39 mM^−1^ in DPPC vesicles. These reductions on the Stern−Volmer constant indicate that the interaction with the membrane occurs with the fluorophores partially exposed to the aqueous environment.

**Figure 5:**
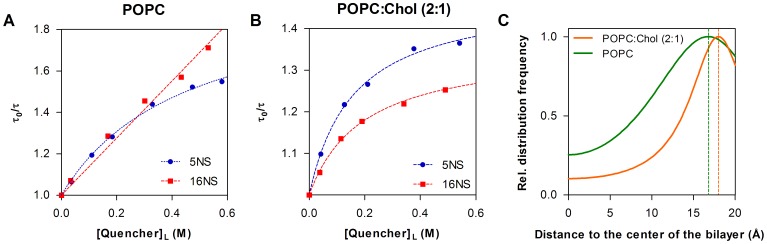
Localization of C34-cholesterol in the bilayer. (**A–B**) Stern-Volmer plots for the quenching of C34-cholesterol fluorescence by 5NS or 16NS in POPC (A) and POPC:Chol 2∶1 (B) LUV, using time-resolved fluorescence measurements. Each point is the average of three independent measures. The dashed lines are fittings of the Lehrer equation (eq. 4) to the experimental data, except for 16NS in POPC (eq. 3). (**C**) Depth of insertion of C34-cholesterol Trp residues in the membrane using SIMEXDA method [36], yielding an average location 16.8 Å away from the center of the bilayer for POPC and 18.0 Å for POPC:Chol 2∶1. Distributions’ half-width at half-height were 8.9 Å for POPC and 6.5 Å for POPC:Chol.

Fluorescence quenching measurements were also used to evaluate the depth of insertion of Trp residues of the peptides inserted in POPC or POPC:Chol (2∶1) vesicles. Stearic acid molecules derivatized with doxyl (quencher) groups either at carbon 5 (5NS) or 16 (16NS) were used. 5NS is a better quencher for molecules inserted in the membrane in a shallow position, close to the lipid-water interface, while 16NS is better for molecules buried deeply in the membrane [36]. Fig. 5A and B shows the Stern-Volmer plot obtained for C34-cholesterol on POPC or POPC:Chol (2∶1), respectively, using the effective concentration of 5NS and 16NS in the bilayer matrix. For POPC, the *K*_sv_ with 5NS was higher (4.43±0.41 mM^−1^, calculated with the Lehrer equation) than for 16NS (1.38±0.052 mM^−1^, calculated with the linear Stern-Volmer equation). In the case of POPC:Chol (2∶1), *K*_sv_ for 5NS was also higher (9.23±0.67 mM^−1^, with the Lehrer equation) than 16NS (6.94±0.44 mM^−1^, with the Lehrer equation). Fluorescence lifetime quenching data enabled the application of the SIMEXDA method [36] to obtain the depth of insertion of the Trp residues of C34-cholesterol (Fig. 5C). A similar mean shallow location, close to the membrane interface, was observed for both lipid membranes. However, in the presence of cholesterol, the depth distribution is narrower, and the location more superficial (more exposed to the aqueous media), in a good agreement with acrylamide quenching data. The location distribution of C34-cholesterol does not differ much from the determined previously for enfuvirtide or T-1249 [10], [35]. As expected, no changes in the fluorescence intensity were detected in the presence of 5NS or 16NS for C34 (data not shown).

### Interaction with Blood Cell Membranes

**Figure 6:**
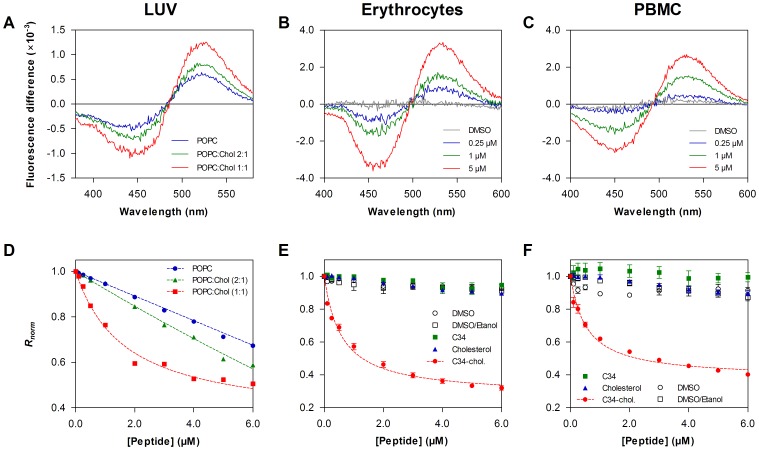
Peptide interactions with di-8-ANEPPS labeled LUV and cell membranes. (A–C) Differential spectra of di-8-ANEPPS bound to LUV (**A**) erythrocytes (**B**) and PBMC (**C**) membranes in the presence of C34-cholesterol and in its absence. Spectra were obtained by subtracting the excitation spectrum (normalized to the integrated areas) of labeled cells in the presence of peptide from the spectrum in the absence of the respective peptide. The shift to the red (decrease in dipole potential) is peptide concentration-dependent. For LUV, spectra traces represent C34-cholesterol 4 µM, in the presence of different lipid compositions: POPC, POPC:Chol 2∶1 and POPC:Chol 1∶1. For cells, spectra traces represent different C34-cholesterol concentrations: 0 µM, 0.25 µM, 1 µM and 5 µM. (**D**) Binding profiles of C34-cholesterol to LUV of POPC, POPC:Chol 2∶1 and POPC:Chol 1∶1, by plotting the di-8-ANEPPS excitation ratio, *R* (I_455_/I_525_, normalized to the initial value), as a function of the peptide concentration. DMSO, cholesterol and C34 (unconjugated) were also tested, as controls, and no significant changes on the dipole potential were observed (data not shown). (**E–F**) Binding profiles of C34-cholesterol, C34 and cholesterol to erythrocytes (**E**) and PBMC (**F**) cell membranes. Controls for DMSO (empty circles) and DMSO:ethanol (empty square) were also performed. Dashed curves represent the fitting to the single binding site equation (eq. 5). Affinity parameters for the cells are indicated in Table 3. Error bars represent SEM, with n = 5 for C34-cholesterol curves in cells and n = 3 for the control curves and LUV.

After the characterization using membrane-model systems, we studied this peptide-membrane interaction in biological settings. We used isolated human erythrocytes and PBMC, labeled with the fluorescent probe di-8-ANEPPS, which is sensitive to the membrane dipole potential [30]. If the peptides interact by inserting or adsorbing on the membrane, it is expected that they change the membrane order to some extent, inducing variations in its dipole potential. These changes can be reported by di-8-ANEPPS excitation spectrum shifts [30], [37], becoming easier to assess in differential spectra (Fig. 6A–C). The fluorescence red shift indicates a decrease in the membrane dipole potential in a peptide concentration-dependent manner. In order to quantify this interaction, we measured the ratio *R* of the intensities at the excitation wavelengths 455 and 525 nm (with emission at 670 nm) for a range of peptide concentrations. *R* is a quantitative descriptor of spectral shifts and, hence, of the relative variation of dipole potential. The membrane dipole potential significantly decreased in the presence of C34-cholesterol (Fig. 6 D–F), in sharp contrast to the unconjugated C34 peptide that had almost no effect. This shows that, in agreement with the model membrane studies, C34 does not have tendency to interact with cell membranes, while C34-cholesterol does. Affinity constants derived from the dipole potential variation curves indicate a much higher affinity when compared with the previously studied HIV fusion inhibitor peptides enfuvirtide and T-1249 (Table 3).

**Table 3:** Antiviral activity and cell membrane interaction parameters compared of HIV-1 fusion inhibitors.

		C34-cholesterol	T-1249	Enfuvirtide (T-20)
IC_90_ (nM)*	JRFL	0.36	8.3	20
	NL4-3	0.09	2.6	6.1
*K*_d_ (µM)	Erythrocytes	0.58±0.049	4.16±0.13†	35.4±1.33†
	PBMC	0.54±0.052	7.71±0.61†	62.2±10.2†

* [11];

† [9].

For the sake of comparison with the data obtained for blood cell membranes, and in order to validate the comparison with previous data, we also labeled lipid vesicles with di-8-ANEPPS (Fig. 6A, D). Lipid vesicles were composed of POPC, POPC:Chol 2∶1 and POPC:Chol 1∶1. In a good agreement with the partition and pressure data, we found that the increase of the cholesterol percentage on the membrane also increased the interaction of C34-cholesterol. Additions of DMSO, cholesterol or C34 were also tested as control and no changes on the dipole potential were observed (data not shown).

## Discussion

In previous works, we showed that the interaction of HIV fusion inhibitor peptides with membranes (using lipid membrane models and blood cells) is a key aspect of their mechanism of action. The membranotropic properties of a peptide can increase its local concentration at the membrane and boost the efficiency of the drug [8]–[10], [29], [35], [38]. In this context, we studied the membrane interactions of the C34 peptide, in comparison with its cholesterol conjugated version, C34-cholesterol, in order to understand the molecular basis of the dramatically increased antiviral efficiency of the conjugate [11].

This study demonstrates the membrane-binding ability of C34-cholesterol. Overall the results obtained from partition, surface pressure, quenching and dipole potential show that the addition of the cholesterol moiety to the C-terminus of C34 renders the peptide membranotropic. The partition coefficients for POPC obtained for this conjugated peptide are of the same order as the previously determined for enfuvirtide and T-1249 (Table 1). In contrast, C34 shows no partition for membranes of this lipid. Enfuvirtide and C34 share a common CHR sequence as a structural domain; however, C34 lacks a putative lipid-binding domain (LBD) that is Trp rich, which may explain its low membrane partition (Fig. 1A) [39]. This is also the case of the LBD-lacking fusion inhibitor peptide sifuvirtide, which also presents residual binding to POPC vesicles and the same preference for DPPC membranes (on the gel phase) [8], [29]. When the interactions with cholesterol-rich membranes were studied, in contrast with the result previously obtained for enfuvirtide [35], membrane partition of C34-cholesterol still takes place and with a relatively high *K*_p_ (Table 1). A similar behavior was found for lipid raft-mimicking membranes (POPC:Chol:SM).

In order to interpret results on POPC:Chol binary mixtures, their phase diagram at 25°C needs to be considered [33]. In the case of pure POPC, the membrane is in the liquid disordered phase (L_d_). For POPC:Chol 2∶1 there is a phase coexistence between liquid ordered (L_o_) and liquid disordered (L_d_) phases, while for POPC:Chol 1∶1 only L_o_ phase occurs (but close to the boundary between L_o_/L_d_ coexistence). Our partition data indicate that C34-cholesterol preferentially partitions to the L_o_ phase, displaying a *K*_p_ three-fold higher than for the L_d_ phase (pure POPC), and an intermediate behavior when both L_d_ and L_o_ phases coexist. Additionally, using di-8-ANEPPS labeled LUV (Fig. 6A, D), we showed that the interaction of the conjugated peptide increases with the amount of cholesterol present in the POPC:Chol binary mixture, suggesting again a preference for cholesterol-rich membranes.

Surface pressure measurements (Fig. 3) showed a higher membrane affinity of C34-cholesterol towards cholesterol and SM-rich membranes, when compared to pure POPC. Taking into account that C34 was unable to induce any change in any of the tested monolayers (data not show) these experiments also showed that the cholesterol moiety boosts the adsorption of C34 specifically on cholesterol-rich membranes. In comparison, enfuvirtide showed much less distinction between the three mixtures tested. Furthermore, regarding the kinetics of the interactions of the three HIV fusion inhibitors in POPC:Chol monolayers (Fig. 3D), we found that C34-cholesterol also exhibit a faster binding kinetic, which could only be assigned to the “membrane anchoring” cholesterol moiety.

This preference of the drug for cholesterol and “lipid raft-like” mixtures is very relevant regarding inhibiting HIV entry. The most important receptors for HIV entry, CD4 and CCR5, were shown to be associated with lipid raft microdomains and DRM (detergent resistant membranes) and this association is important for the binding of the virus to cells [40]. Several studies also demonstrated that depleting cholesterol from the host cells greatly inhibits HIV infection [40]–[42]. Moreover, the substitution of the virion-associated cholesterol by the raft-inhibiting sterol analogues 4-cholestenone and coprostanol was shown to reduce infectivity, in contrast to other raft promoting sterols [43]. Overall, lipid rafts are considered to be essential for the virus entry process [44]. The preferential partition of C34-cholesterol to those domains would make the drug more available at the site where membrane fusion occurs.

Recently, the lipid composition of HIV-1 membrane, constituting its lipidome, has been precisely determined [22]. That allowed us to study lipid vesicles with compositions mimicking the virus envelope. The high C34-cholesterol partition observed for the HIV membrane-like mixture of lipids indicates that the viral membrane itself can also extensively capture and carry the conjugate drug on its surface, enhancing its availability at the required site of action. However, this interaction seems to be sensitive to the presence of DHSM, a lipid found to be unusually enriched at the viral membrane, when compared to the host cell membrane [22]. When present, DHSM decreases C34-cholesterol partition (Table 1). It is tempting to speculate that, in a context of virus and host cell engagement; this may be a factor that shifts the equilibrium of partition towards the cell membranes, where it anchors in the right direction to interact with the transiently exposed gp41 NHR domain.

Regarding the location of C34-cholesterol at the membrane level, our data show that the Trp residues of the peptide insert in the external leaflet of the bilayer, at a shallow position. Previously, the Trp-rich regions of enfuvirtide and T-1249 were also shown to insert superficially in lipid bilayers [10], [35]. As the peptide is conjugated with cholesterol in a defined position, the peptide should adopt a consistent orientation, with the C-terminus anchored to the membrane by the cholesterol moiety (Fig. 1A). This is the proper orientation to align in an anti-parallel fashion with the NHR domain of gp41 when it extends towards the target membrane. The N-terminus, where the Trp residues are located, may interact freely with the nearby lipids, but cannot insert deep in the membrane.

In order to have a better understanding of what may happen in the bloodstream, we also studied the interaction of the peptides with human erythrocytes and mononuclear leukocytes. These peptide drugs must be injected, as oral administration is not feasible. HIV-1 is known to associate to the surface of erythrocytes in circulation [45]–[47], while PBMC are among the virus main targets. C34-cholesterol decreases the membrane dipole potential of the two cell types, indicating that an interaction is occurring (Fig. 6). In agreement with the partition data, C34 alone also does not interact with cell membranes. The same concentration of cholesterol alone is not sufficient to induce a detectable membrane potential change [48], indicating that the potential change is induced essentially by the peptide component of the conjugate. However this change only occurs in the conjugate, emphasizing the role of cholesterol in inducing the membrane partition of the peptide to cell membranes.

Quantitatively, C34-cholesterol was found to have approximately 115 or 14 times more affinity towards PBMC membranes than enfuvirtide or T-1249, respectively. Furthermore, the cell membrane affinities of the different fusion inhibitor peptides (C34-cholesterol>T-1249> enfuvirtide) correlate with the antiviral potency against HIV-1 (Table 3). Regarding the comparison with the *K*_p_ values determined with lipid vesicles, this is not straightforward, as the results obtained for the different compositions tested indicate that the process is lipid composition-dependent. Moreover, the partition is based on tryptophan fluorescence, which regards only the peptide part of the conjugate. However, if we focus on cholesterol-containing mixtures, C34-cholesterol has the highest partition, contrasting with enfuvirtide, with negligible interaction [35]. T-1249 should fall in between, considering its partition to cholesterol-poor domains and ability to adsorb on cholesterol-rich domains [10].

**Figure 7:**
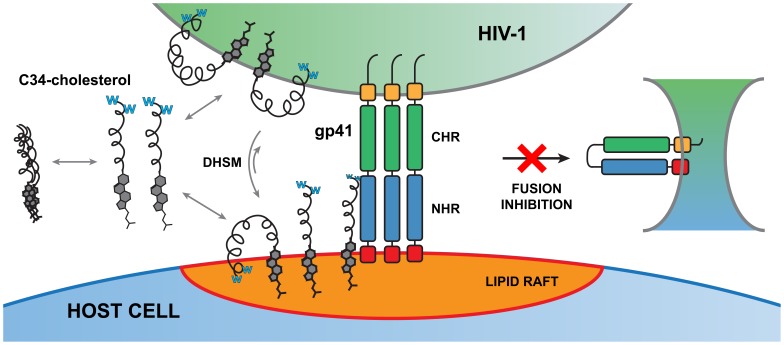
Putative mode of action of HIV-1 fusion inhibitor C34-cholesterol. In aqueous solution, the conjugate may form micro-aggregates when membranes are not present, due to its cholesterol moiety. The drug was demonstrated to partition to cell membranes and virus-like membranes. A preference towards cholesterol and SM-rich compositions was identified. These lipids are characteristic of the membrane microdomains designated as lipid rafts, which usually contain the receptors involved in HIV entry. C34-cholesterol anchors to the membrane *via* its cholesterol moiety and also, with a putative weaker binding, *via* its Trp (W)-rich N-terminal domain. In the context of HIV-1 gp41 engagement with the target cell, a confined space exits between the two membranes. Enrichment in DHSM in the viral membrane decreases the peptide partition, possibly shifting membrane partition equilibrium to the host cell membrane. The drug concentrated in the lipid raft environment may reach its target (gp41) more efficiently than through simple diffusion in aqueous solution. Moreover the anchoring promoted by the cholesterol at the C-terminus brings the peptide into contact in the correct orientation to compete with NHR binding site. This way, gp41 mediated fusion may be inhibited, blocking viral content entry into the cell.

These findings, relating membrane binding to improved antiviral potency of the peptides, indicate that this is a major factor to take into account when rationally designing HIV fusion inhibitor peptides. We demonstrated that C34-cholesterol has membranotropic behavior towards lipid vesicles and strongly interacts with circulating blood cells membranes. HIV fusion inhibitors have a restricted temporal and spatial window of opportunity to bind to their molecular target: the NHR region of gp41 in its extended conformation, when the virus and target cell are closely engaged. This makes aqueous diffusion of the peptide to its target less efficient. It has also been demonstrated that the forces governing protein–protein interactions in a membrane environment are different from those in solution [49]. The ability that these peptides have to bind to cell membranes facilitates the delivery of the peptides to this confined environment, as some peptide is already locally present (Figure 7). In this context, C34-cholesterol should be able to concentrate at the cell surface and also on the viral membrane. In agreement with this, previous results with cells after 48 h of treatment with C34-cholesterol showed the persistence of antiviral activity of residual peptide not cleared by washing steps [11], supporting the idea that cell membranes could work as C34-cholesterol reservoir. Importantly, the concentration effect would be higher on the lipid rafts, where the HIV receptors are present and fusion is more likely to occur [40], [44]. This persistent antiviral activity may be ideal for this drug to be applied as a topical microbicide, for example as vaginal gel, to prevent infection [12]. C34-cholesterol was generally the most active inhibitor when tested in the context of human mucosal tissue explants (colorectal, penile and cervical), when compared to C34, enfuvirtide and T-1249 [12]. The first microbicides formulations against HIV were based on unspecific polyanions to present viral attachment to the cell surface; however, they largely failed in clinical trials [50]. Only recently a trial using a tenofovir gel (a reverse transcriptase inhibitor) showed 39% reduction on HIV-1 infection [51]. Using an entry inhibitor such as C34-cholesterol could prevent the viral entry in the first encountered immunological cells of the mucosa, blocking the virus at the earliest stage of infection.

Ultimately, the membrane may act as a “catalyst” to the binding reaction between gp41 and the peptide inhibitors, as it has been postulated in other receptor-ligand scenarios in membrane environments [38]. Thus, in this case, the marked increase in antiviral efficiency of C34-cholesterol in relation to C34 (and other fusion inhibitors) correlates with its higher affinity towards model and blood cells membranes.
